# A case study of reservoir characterization and modeling for tidal-dominated estuary reservoir in Ecuador

**DOI:** 10.1038/s41598-025-03591-7

**Published:** 2025-06-04

**Authors:** Cheng Jiwei, Zhang Kexin, Zhang Chaoqian, Li Yunbo, Feng Taoran, Liu Jian, Xu Xianglin, Ma Zhongzhen

**Affiliations:** 1 PetroChina Research Institute of Petroleum Exploration and Development, Beijing, China; 2 CNPC Andes (Ecuador) Company Limited, Quito, Ecuador; 3 China National Oil and Gas Exploration and Development Corporation, Beijing, China

**Keywords:** Geophysics, Fossil fuels, Solid Earth sciences

## Abstract

The Oriente Basin, as part of the retro-arc foreland basin system, develops tidal-dominated estuary, where the LU layer exhibits complex sedimentary characteristics in lateral that brings great uncertainty to reservoir characterization using only wells. As a result, it is essential to investigate the sedimentary characteristics of the tidal-dominated estuary by incorporating seismic data. First, four electrofacies are established to characterize the logging response of various lithology combinations in an estuary setting. The seismic facies are then studied by establishing the relationship between seismic waveforms and logging response. Finally, a reservoir model of the LU layer is built by quantitatively characterizing the spatial distribution of sedimentary facies based on the electrofacies and seismic facies. Bottom-water reservoirs are reinterpreted as a combination of the upper edge-water reservoir and the lower bottom-water reservoir based on the characterization results, which are verified by the new drilling wells.

## Introduction

The tidal-dominated estuary sedimentary system is a primary reservoir for numerous oil fields worldwide and has demonstrated significant promise in oil exploration in recent years, such as the Athabasca oil sandstone layer in the Lower Cretaceous McMurray Formation of the Albert Basin, Canada^1^, M1, U and T sandstone layers in the Upper Cretaceous Napo Formation of the Oriente Basin, Ecuador^2^, Oficina sandstone layer in the Miocene of the Venezuela Basin, eastern Venezuela^3^, Springhill sandstone layer in the Lower Cretaceous of the Austral Basin, Argentina^4^, Upper Cape Hay sandstone layer in the Late Devonian–Carboniferous age of the Joseph Bonaparte Basin, Australia^5,6^, and the Kalpingtage sandstone layer in the Silurian of the Tarim Basin, China^7^. The dual hydrodynamics of tides and waves affect tidal-dominated estuary sedimentary environments differently than those found in typical fluvial and deltaic sedimentary environments. This results in multi-scale heterogeneity, complex lithology, and architecture variation, making tidal-dominated estuary sandstone difficult to study and resulting in scarce relevant research^8,9^. The deterministic method relying only on wells cannot meet the demand for characterizing reservoir properties precisely in such circumstances^10^. These challenges place high demands on fine-scale reservoir characterization, particularly when integrating with seismic data. The final model will reflect the actual sedimentary geological features, and the variogram values used for geological modeling will be more accurate.

Reservoir characterization is a crucial step in developing, monitoring, and managing a reservoir to optimize production. An electrofacies is a collective association of log responses that are connected to physical properties of rocks either implicitly through interpretation or explicitly linked to a lithology database. Identifying electrofacies usually aims to correlate them with lithofacies identified in the core or an outcrop. The goal of seismic facies analysis is to identify all seismic parameter variations within a depositional sequence’s stratigraphic framework in order to determine lateral lithofacies and fluid type changes. Although identification of electrofacies and seismic facies is significant and indispensable steps in reservoir characterization, they are still insufficient for the LU layer in Ecuador. Presently, the further reservoir characterization method involves incorporating all available information, which includes core data, well log data, and seismic dataset, into a reservoir model to predict the distribution and properties of subsurface reservoirs, ultimately building a three-dimensional quantitative reservoir model^11–17^. This process synthesizes multiple disciplines, including logging, sedimentology, and geophysics, to interpret rock facies and achieve calibration based on data from wells, core samples, seismic signals, and geological understanding, and establish a reservoir geological knowledge database.

Reservoir modeling is an essential step to quantitatively characterize subsurface properties and serves as the foundation for reservoir simulation in the development and production of oil fields. Based on the accurate reservoir model, sweet spots or regions with greater porosity, permeability, and hydrocarbon saturation can be acquired. Operators can reduce the risk of dry holes and improve their chances of finding productive reservoirs by focusing their drilling efforts on these zones. Various geological modeling techniques have been developed and made great progress to meet the high requirement^14,18–24^. Combining the reliable representation of a reservoir that incorporates its petrophysical, geological, and geophysical characteristics with advanced geological modeling methods, an accurate three-dimensional reservoir model can be created in a mature oil field.

The study area in Ecuador, exhibiting the sedimentary characteristics of a tidal-dominated estuary, contains several oilfields. It is crucial to perform fine reservoir characterization and potential analysis in this region for the development of oilfields. The LU layer is the primary prospective successor target among the three sets of oil-bearing reservoirs found in the M oilfield, which has undergone successive drilling in recent years. The complex sedimentary characteristics of the estuary reservoirs in this area and the limited understanding of the interlayers using only wells have brought great uncertainty to the current reservoir fine characterization. Therefore, it is essential to investigate the tidal-dominated estuary sedimentary characteristics and to characterize the sandstone spatial distribution and the interlayer by incorporating seismic data. First, the location, tectonics, and sedimentary characteristics of the study area are briefly described. Electrofacies are then identified and established using core and well log in the specified regional context in order to ascertain the logging response characteristics of various electrofacies. Next, in order to conduct the seismic facies analysis and describe the reservoir distribution in the spatial domain, the link between seismic waveforms and petrophysical parameters is established. Finally, based on the electrofacies and seismic facies, we perform sedimentary facies characterization and modeling to establish a reservoir model of the LU layer in the study area and to clarify the future potential for the reservoir.

## Geological setting

### Location of the study area

**Fig. 1:**
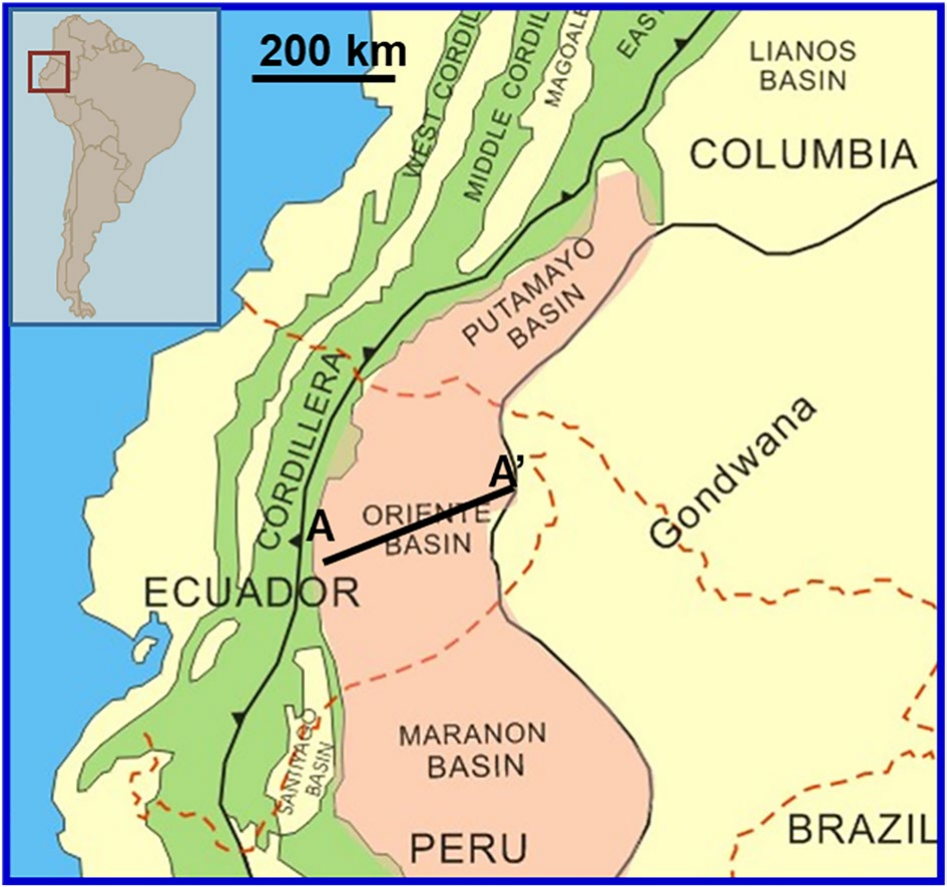
Simplified regional geology of northwestern South America showing the distribution of Oriente Basin in Ecuador.

The most significant oil-bearing basin in Ecuador is the Oriente Basin, which is located in the Amazonian Plain of eastern Ecuador and is a part of the Putumayo-Oriente-Maranon oil-rich foreland basin chain in South America^25,26^. The basin is asymmetrically dipping with nearly north–south trending, steep in the west and gentle in the east, and covers an area of about 10 × 10^4^ km^2^. The basin is located in the transition zone between the tectonically active Cordillera and the stable Brazil-Guyana shield (Fig. 1). The total recoverable resources of the basin are about 15 ~ 17 × 10^8^ tons^27^, which has a large potential for exploration and development.

### Tectonics

**Fig. 2:**
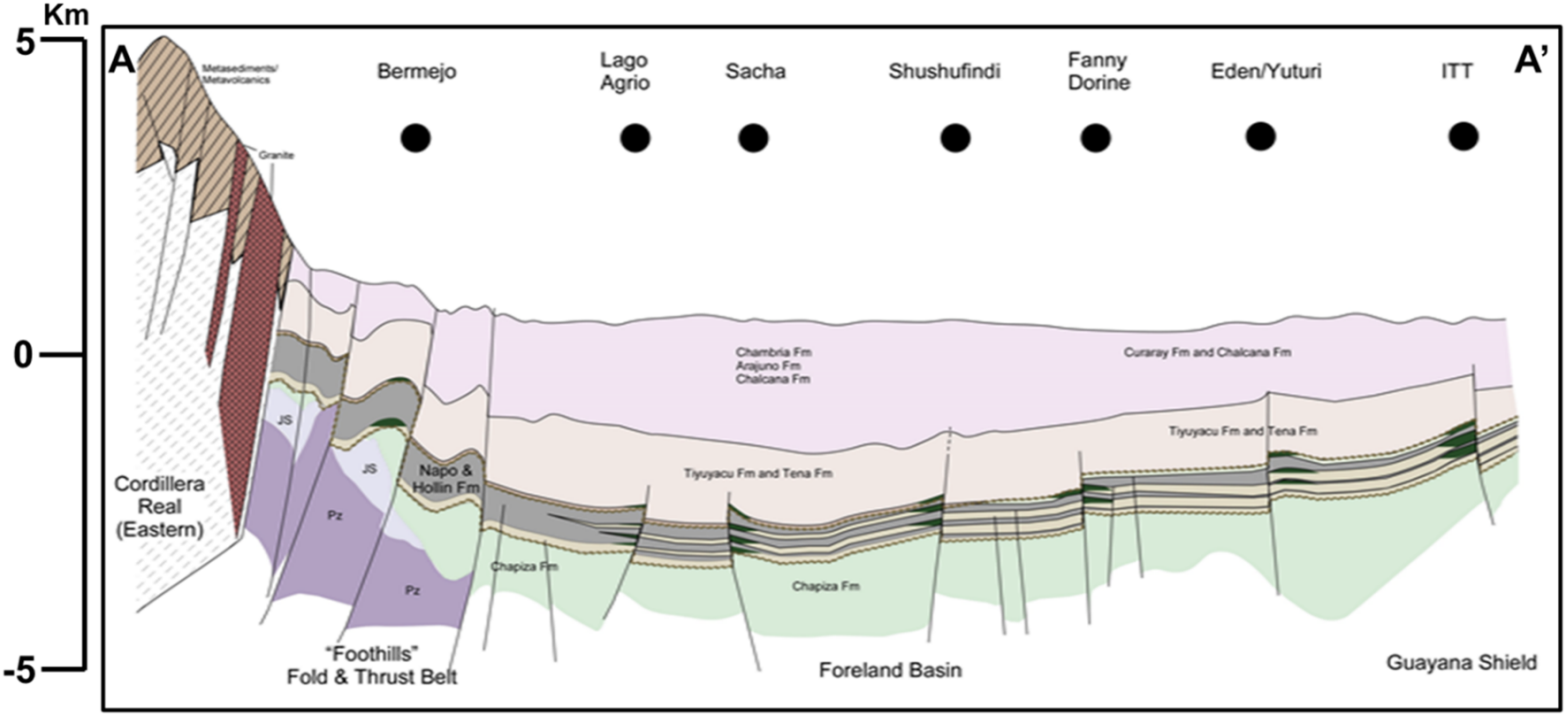
Structural cross-sections across the Oriente foreland basin system.

Oriente basin, as the foreland basin, overlaps on the rifting basin^27^, where the study area is located in the slope among the strike-slip fault system of line AA’ from Fig. 1 as shown in Fig. 2. The tectonic evolution of the Oriente Basin has gone through three main stages since the Paleozoic period^28,29^: the Paleozoic passive continental margin stage, the Triassic-Cretaceous rift basin stage, and the late Cretaceous-present foreland basin stage. First, in the Paleozoic passive continental margin stage: the basement of the basin consists of Precambrian magmatic rocks and metamorphic rocks, where the Paleozoic stratigraphy involves two stratigraphic sequences, the lower one is the late Silurian-early Devonian Pumbuiza Formation, consisting of moderately metamorphosed limestone, shale, silty mudstone and sandstone, and the upper one is the Macuma Formation, composed of thin laminated limestone and mudstone, which is deposited on the continental shelf of the passive continental margin in shallow marine carbonate rock. Second, Triassic-Cretaceous rift basin stage: During the Triassic period, the eastern boundary of the Pacific plate was converted from a passive continental margin to an active continental margin and developed nearly north–south normal faults. A series of north–south oriented grabens developed, which was filled with shallow marine-terrestrial sedimentary and acidic igneous rock. Then, the Cretaceous post-rift depression, including the Hollin Formation and the Napo Formation, overlying the rift system, which exhibits fluvial facies, interactive marine and terrestrial facies, and neritic shelf facies, and the main oil-bearing sandstone are M1, U, and T layers of the Napo Formation. Finally, the late Cretaceous—present-day foreland basin stage: during the late early Cretaceous—late Cretaceous period, marginal sea and four major basins are developed on the east side of the Andes. Since the end of the Cretaceous, at least three tectonic accretions occurred, resulting in the uplift of the Eastern Cordillera—Andes orogeny, which led to the evolution of the Oriente Basin into a foreland basin.

### Stratigraphic and sedimentary characteristics

**Fig. 3:**
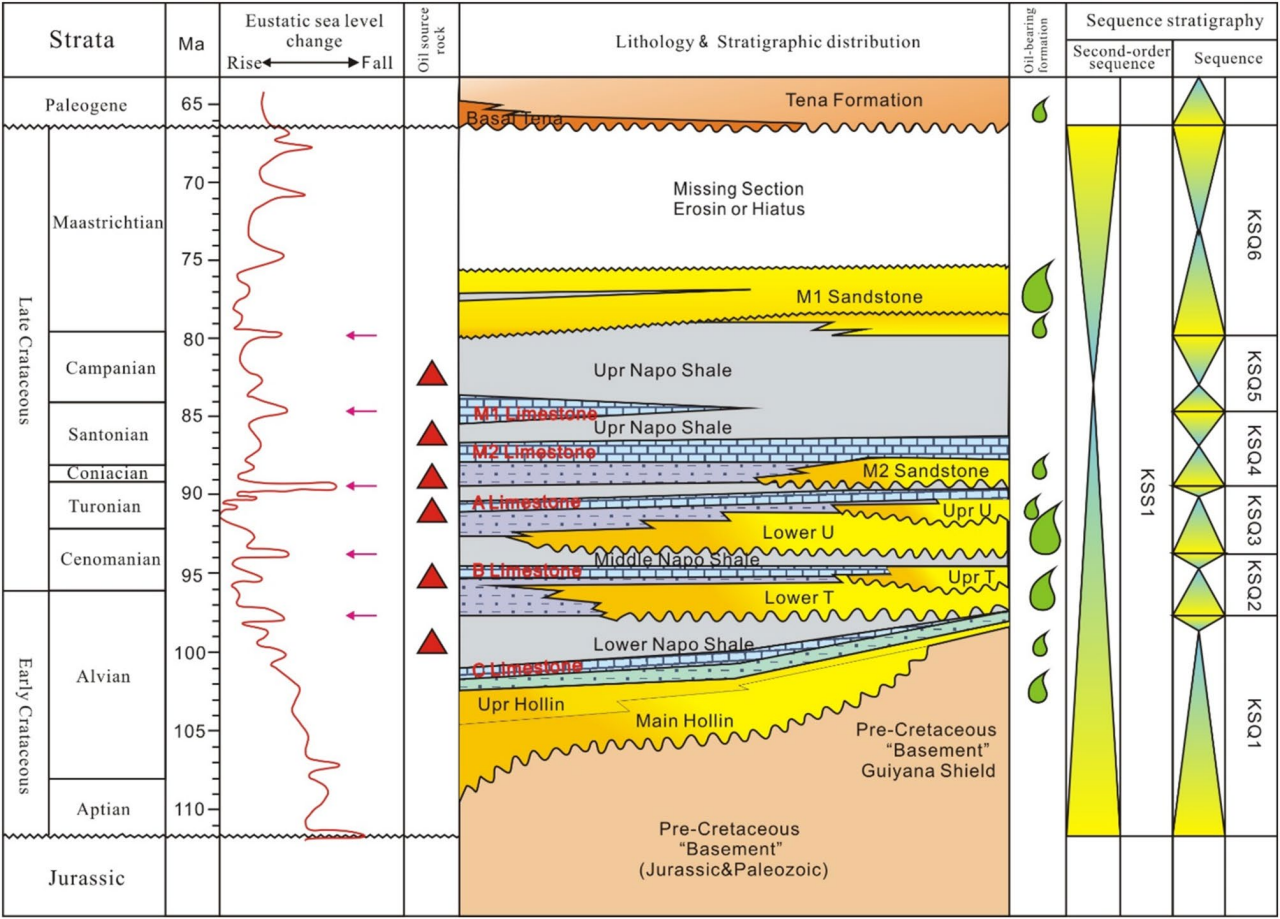
Stratigraphic column of the Oriente Basin (originally produced by^42^). Magenta arrows represent the five transgressions in Napo formation. Size of the water-droplet logos for ‘oil-bearing formation’ column indicate reservoir volume.

The Cretaceous exhibits interactive marine and terrestrial sedimentary, where the main hydrocarbon source rock is marine shale and asphaltene carbonate rocks in the Upper Cretaceous Napo Formation^30^. The main reservoirs are the M1, U, and T sandstone of the Napo Formation as shown in Fig. 3. Shale, coal, and carbonate rocks are local seals in the Tena Formation. The trap finally formed in the early Cretaceous-Palaeocene.

Metamorphic basement and sedimentary infill are the two sets of stratigraphic sequences found in the Oriente Basin, based on the outcrop data analysis and well drilling findings^31^. From bottom to top, the basin fill can be separated into three sections: the pre-Cretaceous sedimentary layer, overlying strata with an unconformity contact, which is overlain by the interactive terrestrial and shallow marine Cretaceous sedimentary layer, and late-Cretaceous terrestrial foreland layer. The Lower Napo Formation develops gray-black rich organic matter shale, and oil is found in the shale at the bottom of the Napo Formation with a large thickness (15–20 m), stable distribution at moderate depth (2,000–3,000 m) throughout the basin. The formation exhibits interbed lamination of sandstone-mudstone interlayers, which are verified by abundant research data (most of the drilling wells in different blocks have encountered this layer), indicating that the Napo Formation is a favorable reservoir unit.

According to the core and well log, the Napo Formation in the study area experienced five transgressions (magenta arrows in Fig. 3) during its deposition. The middle and lower parts of the first, second, and third transgressions developed quartz sandstone, while shale, mudstone, and limestone deposited at the top. The fourth and fifth transgressions deposited marlstone, limestone, mudstone, and shale, which are characterized by the deposition of clastic and carbonate rock^32^. Based on the results of the time–temperature relationship analysis, the source rock in the Oriente Basin did not begin to generate and migrate hydrocarbons until the Miocene^33^. Statistical analysis of the oil abundance in discovered reservoirs shows a sufficient supply of hydrocarbons in the area. Meanwhile, from the viewpoint of tectonic, it is a favorable area in the path of hydrocarbon migration, and the hydrocarbon source rock is relatively good. Reservoir property analysis shows that the Napo Formation is the most important oil-bearing layer in the area, which mainly exhibits estuary facies, including three sandstone (M1, U, and T) oil-bearing layers. Among them, the U sandstone has a large variation in grain size, with tidal channel and sand bar sedimentary characteristics, with porosity of 10–25%, permeability of 100 × 10^–3^–6000 × 10^–3^ μm^2^, and an average total thickness of 45.8 ft.

## Methods

**Fig. 4:**
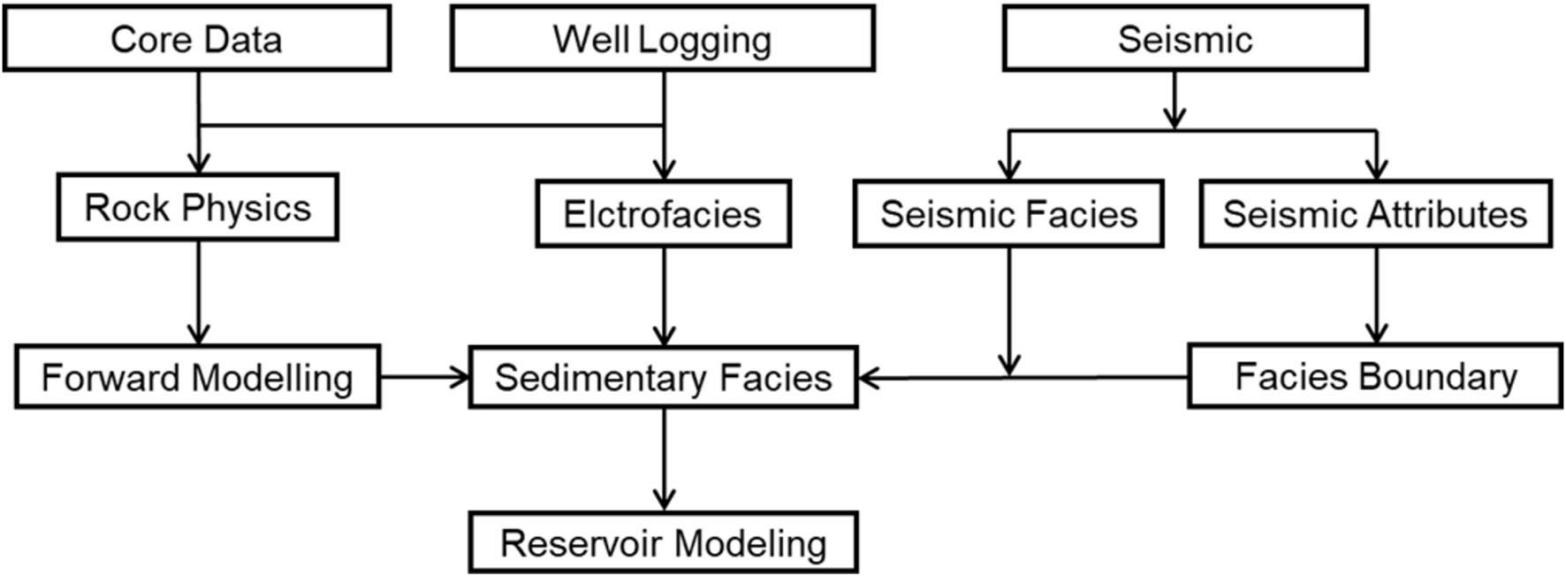
Workflow of reservoir characterization, including the identification of electrofacies, seismic facies and sedimentary facies.

First, a comprehensive analysis of the well log’s properties is conducted using both core and well log. Using the morphology, amplitude, and rock-electricity relationship characteristics of the well log and the core data, the relationship between the lithological combination and the electrofacies model is categorized and summarized. The pattern variation of the well logs is also identified, and the electrofacies model is established. This allows for the analysis and interpretation of the sedimentary features of the layers for the non-cored wells. Then, combined with seismic data, seismic facies analysis is performed in geological interpretation. This can be used to ascertain the lateral distribution range and sedimentary facies of sandstone reservoirs, as well as to examine the history of geological development for sedimentary basins and the viability of industrial exploitation for the exploration area. Lastly, the spatial distribution of reservoir properties in the subsurface reservoir can be quantitatively described by combining geology, logging, and seismic data. Workflow of research is shown in Fig. 4.

### Electrofacies model

The well log is the basic and direct information of the sedimentary microfacies identification for stratigraphy and sandstone distribution analysis. The sedimentary environment can be interpreted by using well log to extract logging information such as amplitude, morphology, contact relationship, and combination of features that reflect stratigraphic features, lithology, physical properties, and sandstone thickness. Therefore, the link between the core facies and electrofacies may be precisely established to reflect the sedimentary facies under the calibration of core and sedimentary features. The major steps involved in electrofacies classification are as follows:

- Conventional well log, such as gamma ray (GR), resistivity curves are calibrated using core samples. The geological characteristics of the estuary reservoirs in the M oilfield are examined.
- The sensitive parameters of reservoirs are determined and electrofacies models are established using chosen sensitive curves.
- Data processed by Principal Component Analysis (PCA) can replace multiple types of conventional well log with the first several principal components and can represent more than 90% of the original information, thereby simplifying the data structure^34^.
- After PCA, K-means is applied to group data points with similar conventional well log into one category, conduct data mining and elelctrofacies calibration, and establish an electrofacies database by minimizing Euclidean distance cluster:
  $$C\left( l \right) = \arg \mathop {\min \left[ {\sum\limits_{r = 1}^{p} {\left| {xir - xjr} \right|^{2} } } \right]^{\frac{1}{2}} }\limits_{1 \le l \le k} ,i = 1,2, \ldots N$$

where *xir* is the r-th feature parameter of the i-th data point, *C(l)* is the set of data points included in the *l* category.

**Fig. 5:**
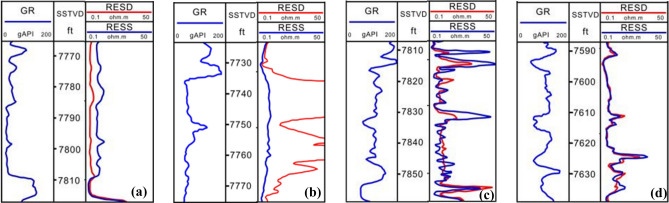
Electrofacies Model of complex sand bodies in the study area. (**a**) Tidal channel, (**b**) Tidal sand bar, (**c**) Tidal sand flat and (**d**) Mixed sand flat.

Based on the core analysis result, in this study, the characteristics of four electrofacies can be established by analyzing the well log characteristics of wells as shown in Fig. 5.

#### Tidal channel

The logging morphology is dominated by box-type or bell-type, with obvious scour filling at the bottom. The gamma-ray (GR) curve exhibits high amplitude, medium-thick thickness, and smooth electrofacies characteristics with abrupt variation at the bottom and gradual variation at the top.

#### Tidal sand bar

It consists of medium and fine sandstone, with the development of trough cross-bedding, planar cross-bedding, and bidirectional cross-bedding. Tidal sand bar develops at the mouth of tidal-dominated estuary, and suffers from the dual control of tide and wave, so the mudstone content is extremely low, which shows very low amplitude in the GR curve, low compressional slowness, high resistivity with a box-type or funnel-type.

#### Tidal sand flat

It consists of fine sandstone interbedded with 10% to 15% mudstone, with parallel bedding and planar cross-bedding. Sand flat characterizes low amplitude serrated in the GR curve, low compressional slowness, and high resistivity with funnel-type or serrated type.

#### Mixed sand flat

The sandstone and mudstone mixed sand flat consist of fine sandstone and silt mudstone interlayers, with parallel bedding and lenticular bedding, representing lower energy conditions. The mixed sand flat in the study area shows a medium amplitude GR curve, medium compressional slowness, and low resistivity with serrated type.

### Seismic facies model

It is important to establish the relationship between seismic data and petrophysics by analyzing seismic response from forward modeling. To classify seismic facies, we apply a typical machine-learning workflow^35^:

*Feature extraction* Seismic attributes are typically extracted by nonlinear transformations of the original seismic data by:

$${\text{X}}_{{\text{i}}} = {\text{T}}_{{\text{i}}} \left( {{\text{X}}_{0} } \right)$$

where X_0_ is the original data, Ti are the transformations, and X_i_ are the resulting seismic attributes that were normalized.

*Training* Classification problems often become easier when we transform a feature into a high-dimensional space. Kernel functions allow an implicit use of these high-dimensional spaces by calculating inner products between feature pair images:

$$K\left( {{\text{x}},x^{\prime}} \right) = \left\langle {\varphi \left( x \right),\;\varphi \left( {x^{\prime}} \right)} \right\rangle$$

- Testing. Cross-validation are performed to quantify the model performance.
- Model selection. Select model based on generalization performance of trained models on test data.
- Application.

Based on the petrophysical analysis of available well data, the basic petrophysical properties of each layer in the area are characterized and summarized. The thickness of sandstone for the LU layer in the study area is generally about 30 ft, whereas the thinnest thickness of sandstone is about 9–18 ft. The average bulk density of sandstone and mudstone are 2.30 g/cm^3^ and 2.45 g/cm^3^, respectively. Compressional slowness of sandstone and mudstone are 82 μs/ft and 90 μs/ft, respectively. The main frequency of seismic data in the study area is 40 Hz. The characteristics of the seismic waveform for the sandstone with different combinations in the vertical direction and lateral direction are tested using forward modeling. First, the correspondence between the thickness of interlayer mudstone and the seismic waveform is tested in the vertical direction. Then, the correspondence between the lateral stacking extent of sandstone and the seismic waveform is tested in the lateral direction.

**Fig. 6:**
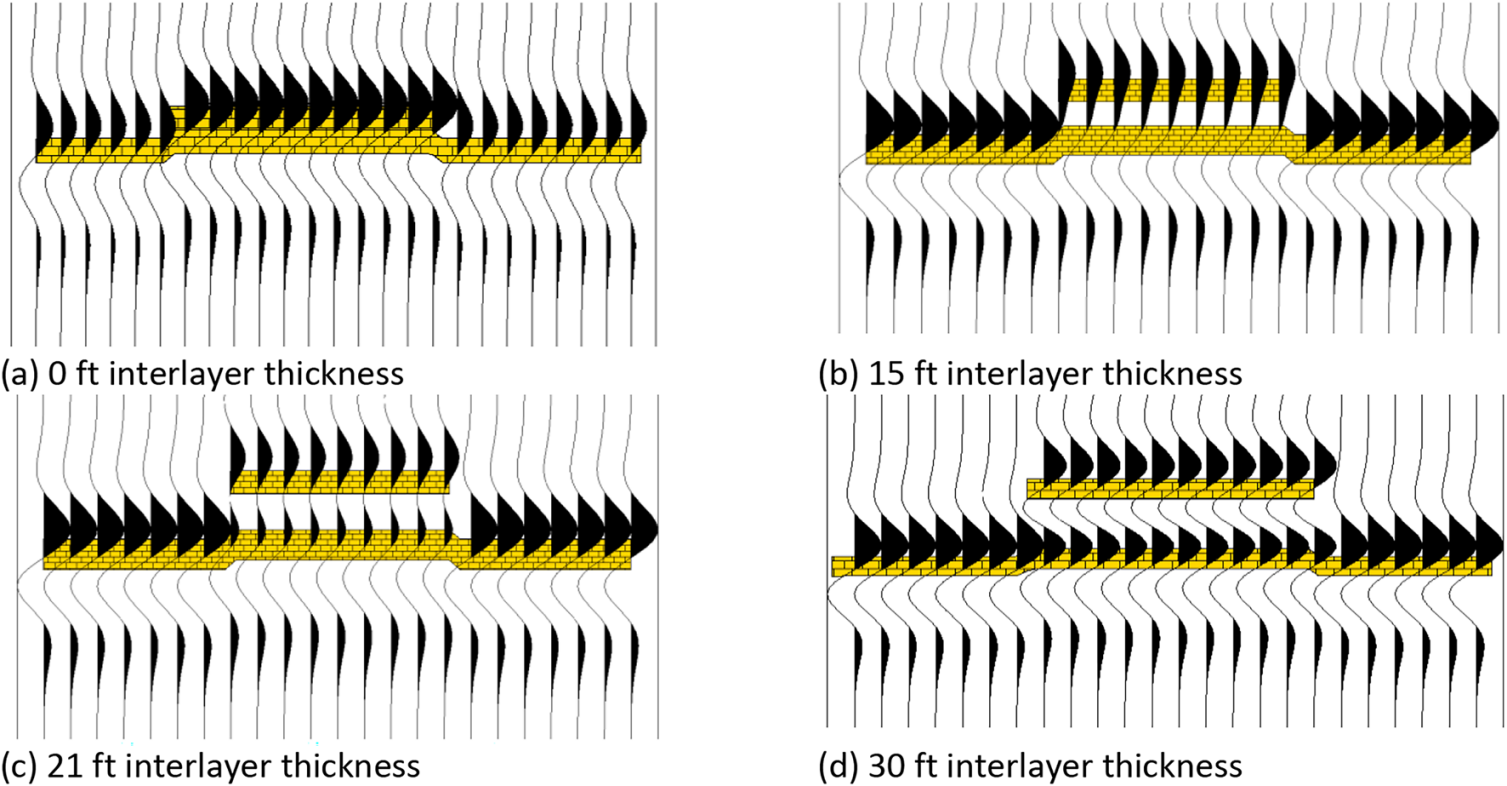
Different interlayer mudstone thicknesses with their seismic response characteristics.

In Fig. 6, it is illustrated that with the increase of interlayer mudstone thickness in the vertical direction, a complex wave, compared with lateral waveforms, is first generated, then the wavelength of the complex wave increases, and after that, a complex wave splits into two high-frequency wave peaks with weak amplitudes, and finally, the amplitude of the wave trough increases between two separate wave peaks. Meanwhile, the stacking of seismic waveforms can be separated in the lateral direction.

Within the isochronous stratigraphy, the variation of the seismic waveform can reflect different lithological combinations, which are expressions of sedimentary or seismic facies, that is, seismic waveforms can be used to reflect the variation of lithology and sedimentary environment in lateral since similar sedimentary environment has analogous sedimentary characteristics. Waveform inversion, utilizing the basic principle of sedimentology, can analyze the high-frequency structural characteristics of the vertical lithological combination for the reservoir, and makes full use of the lateral variation of seismic waveforms to reflect facies characteristics of the reservoir, which can introduce the constraint of facies into the study. Then, the sedimentary rhythms of each layer can be well recognized in the profile, and the sandstone or sedimentary facies boundary can be seen laterally.

**Fig. 7:**
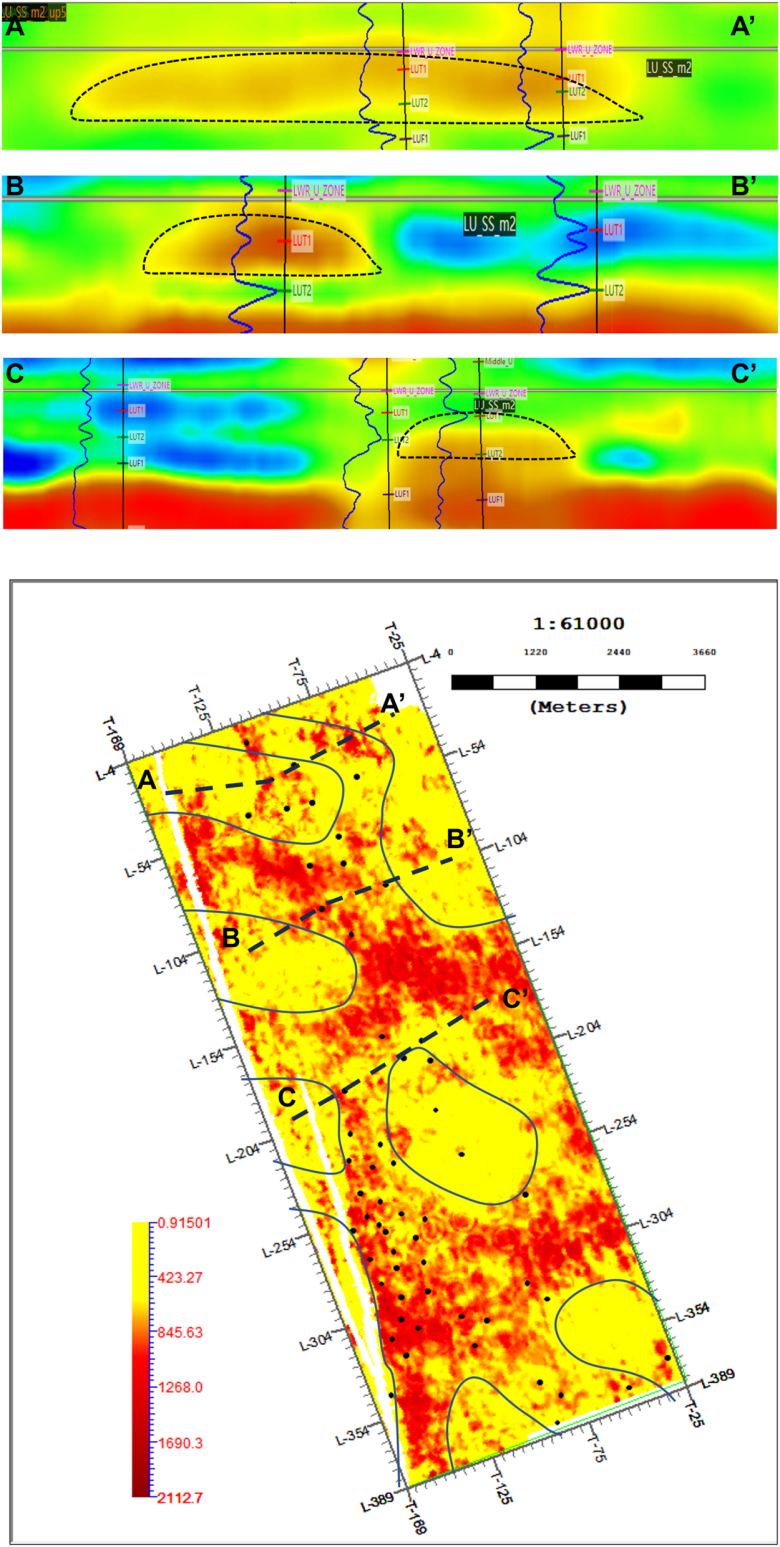
Waveform inversion results and interpreted wells.

The inversion result profile is compared and analyzed with the lithology of a single well, and the results are shown in Fig. 7 that the inversion profile is in good agreement with the interpreted lithology of logging interpretation. The spatial distribution of sandstone between wells is also well-matched with the inversion results.

### Combination of electrofacies and seismic facies

Electrofacies are derived from conventional log data which rely on the radioactivity of subsurface rocks, while seismic facies are generated from seismic data that depend on the elastic properties of the rocks. It is of great significance to establish the relationship between seismic data and petrophysics and incorporate both to gain a more comprehensive understanding in a workflow. The key processes for integrating and calibrating electrofacies and seismic data are as follows:

- *Vertical alignment* Stretching reservoir intervals to match key horizons, ensuring that correlative markers are aligned with corresponding seismic horizons and stratigraphic grid layers. Cross checking across multiple wells is also executed to maintain reasonable interval velocities.
- *Stratigraphic grid construction* Existing mapped horizons are leveraged to define the grid boundaries and internal geometries. Stratigraphic grids are constructed in the time domain that has a resolution comparable to that of the seismic data.
- *Resampling* Resample electrofacies onto stratigraphic grid in order to calibrate the seismic facies in the model.
- *Electrofacies—seismic facies calibration* Establish probability matrix between seismic facies and electrofacies to characterize the relationship:
  $${\text{P}}\left( {E_{i} = e_{i} \cap S_{j} = s_{j} } \right) = \frac{{{\text{P}}\left( {\left. {S_{j} = s_{j} } \right|E_{i} = e_{i} } \right)}}{{{\text{P}}\left( {E_{i} = e_{i} } \right)}}$$

where electrofacies are represented by $\left\{ {e_{1} ,e_{2} \ldots e_{n} } \right\}$, random variables $E_{i}$ such that $E_{i} = e_{i}$ for $i = 1, \ldots ,n$, which means $E_{i}$ is a random variable that takes on the value of a particular electrofacies. Seismic facies are denoted by $\left\{ {s_{1} ,s_{2} \ldots s_{m} } \right\}$ and are represented by the random variable $S_{j}$.

### Sedimentary facies model

Despite electrofacies and seismic facies being two different types of geological data that are measured through different methods, inconsistencies between the two data sets can gain a more comprehensive understanding of subsurface geology. In addition, it is important to consider the limitations of each data set. For example, electrofacies data may be more sensitive to changes in fluid content and porosity, while seismic facies data may be more sensitive to changes in density and velocity. By recognizing the strengths and weaknesses of each data set, it may be possible to develop a more accurate interpretation of subsurface geology. Above all, it is important to use multiple pieces of evidence from other sources, such as core samples, well logs, and seismic signal analysis, to support interpretations of subsurface geology and identify the inconsistencies between the two datasets can lead to a more comprehensive understanding of subsurface geology.

**Fig. 8:**
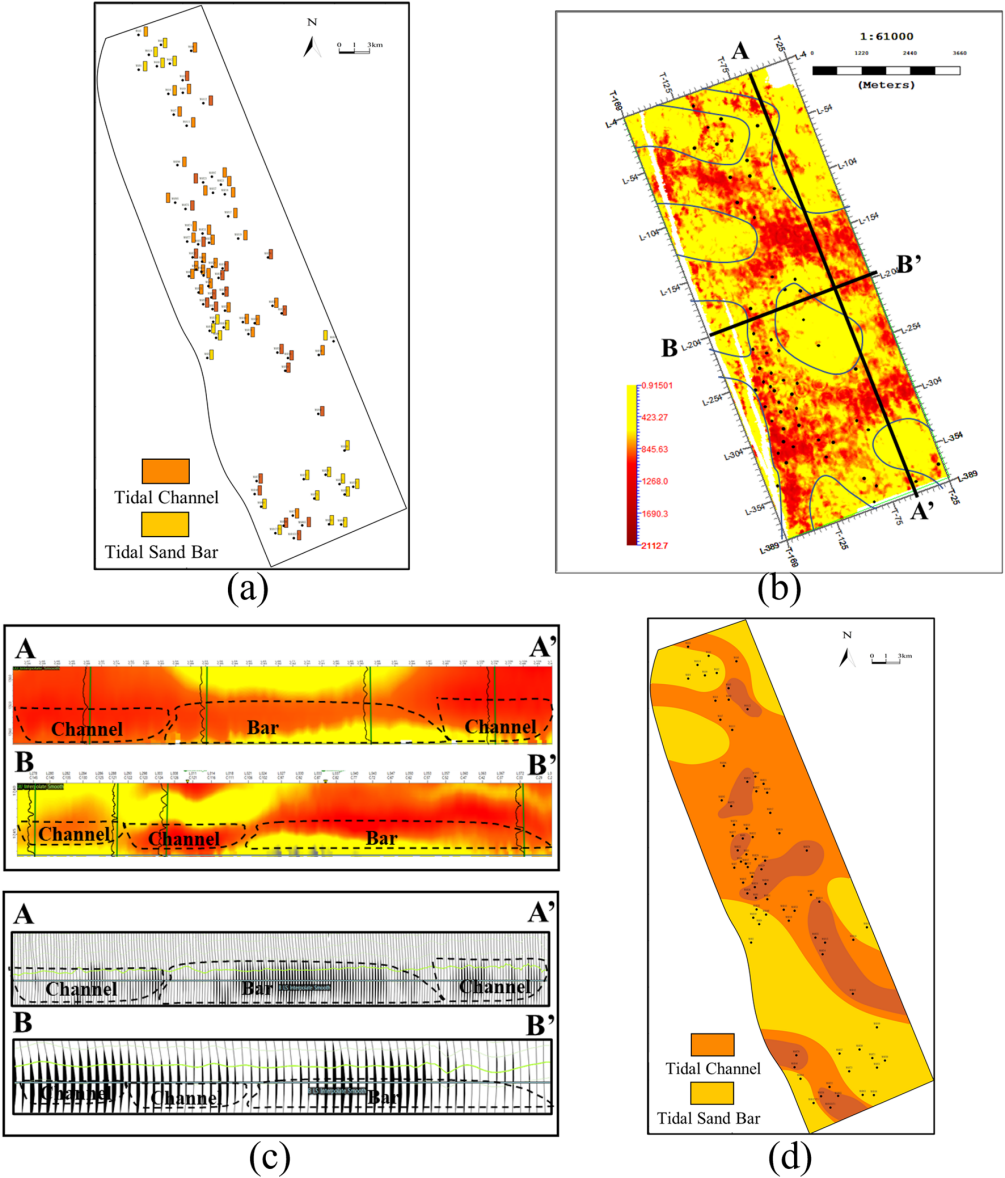
Sedimentary facies characterization and distribution. (**a**) Interpreted electrofacies at well locations. (**b**) Waveform inversion result. (**c**) Facies boundaries from waveform inversion. (**d**) Sedimentary facies distribution.

Combined electrofacies from sandstone characteristics in vertical direction and seismic facies in lateral direction with trend mapping from seismic attributes and facies boundaries from the inversion profile, a reliable study workflow of sedimentary facies can be established. As shown in Fig. 8a, the interpreted electrofacies at well locations is determined based on core data and well log, and the approximate distribution range of sedimentary facies for the target layer can be roughly determined. However, in areas without well control, multiple possibilities and uncertainties exist when interpreting facies. Therefore, trend control using seismic attributes is imperative. The preferred seismic attribute can express lithological combinations and distribution characteristics of the target layer. Figure 8b shows a selected seismic attribute slice, calibrated using interpreted log-based lithology. The red areas in the attribute slice show good consistency with the thick sandstone intervals. As shown in Fig. 8c, seismic facies features are distinctive in the profile, thus seismic facies boundaries can be distinguished in the inversion profiles. Finally, sedimentary facies distribution can be further defined and shown in Fig. 8d with the constrained inversion profile based on the identification result of sedimentary facies using only wells and seismic attributes. The predicted sedimentary facies result reflects facies distribution from interpreted well log and boundary from waveform inversion results, respectively.

## Results

The sandstone of the Lower U could be subdivided into LU_T and LU_F. The LU_T mainly develops thin tidal sandstone interbedded with mudstone, while the LU_F mainly consists of thick fluvial quartzose sandstone. The LU_T layer develops tidal flat deposition, the average thickness of sandstone is 15.2 ft, and the average porosity is 18.7%; the LU_F layer develops multi-phase tidal channels, the average sandstone thickness is 87.3 ft, the average porosity is 20.3%, and the sandstone layer is laterally continuous. Four architectural types are characterized in the vertical direction: (1) upper sand bar with lower sand bar, (2) upper sand bar with lower channel, (3) upper channel with lower channel, and (4) upper channel with lower sand bar. In detail, the LU layer can be divided into upper LU_T and lower LU_F and analyzed architectural characteristics of the inner composite sand-bodies.

### Architecture analysis of LU_T layer composite sand-bodies

**Fig. 9:**
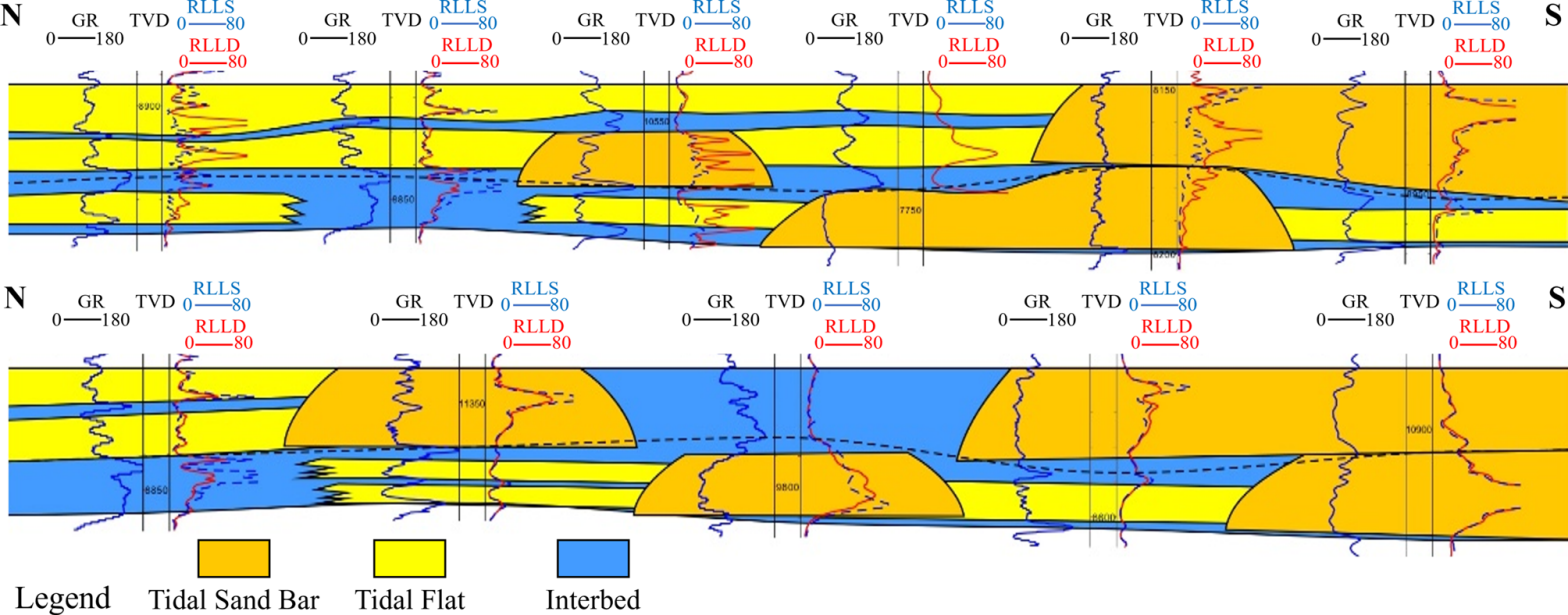
Architecture analysis of LU_T layer composite sand-bodies.

LU_T layer is a composite sand-bodies where tidal flat and tidal sand bar are vertically separated, and the tidal sand bar is tangentially stacked lateral, as shown in Fig. 9. The vertical interface of the architecture characterizes vertically separated interlayers with strong vertical blocking ability. The thin sandstone in the southern part of the study area is well developed, which is better than that in the northern part of the study area.

### Architecture analysis of LU_F layer composite sand-bodies

**Fig. 10:**
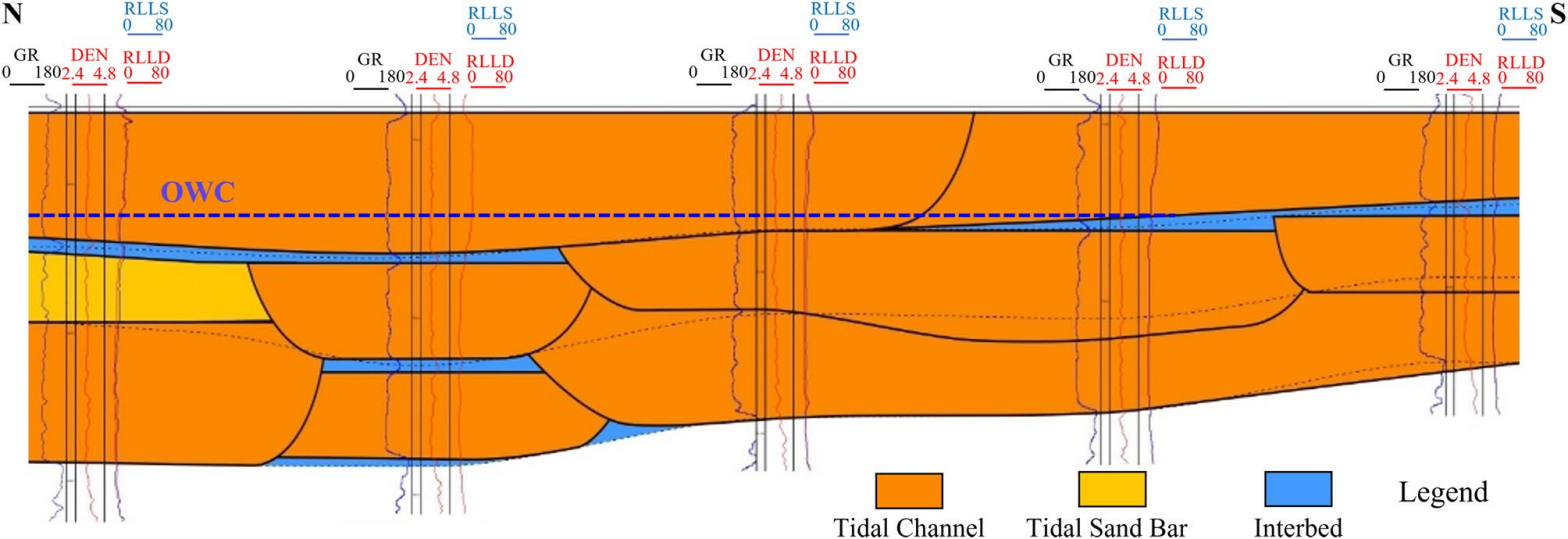
LU_F layer composite sand-bodies.

LU_F layer is a composite sand-bodies where a tidal channel and tidal sand bar are superimposed on each other as shown in Fig. 10. The vertical interface of the architecture is visible as separated interlayers and partially stacked interlayers with strong and weak vertical blocking ability, respectively. The vertical sandstone connectivity in the southern part of the study area is better than that in the northern part with good separated interlayer development and shown as a bottom-water oil reservoir. The well log interpretation results show a distinct oil—water contact, and the bottom water bodies indicated by the interpreted results of multiple wells confirm the existence of a large—area water body at the bottom. Production data also verifies that the bottom water body has sufficient energy, and after production starts, the water—cut rises rapidly while the pressure remains stable.

Based on the statistical analysis of the interlayers in this area, the interlayers can be divided into three categories. From Type I to Type III, the vertical blocking ability weakens. Type I: There is a stable mudstone or silty mudstone separating two single sand bodies, and the thickness is generally greater than 0.5 m. It reveals that there is a low-energy sedimentation period during the high-energy development period of the two single sand bodies in the composite sand body. It is jointly controlled by the distribution ranges of the upper and lower sand bodies, and has a strong blocking ability. Type II: The thickness of the interlayer between the two single sand bodies is very small, generally less than 0.5 m, which shows that the upper channel sand body has eroded the underlying mudstone interlayer. Vertically, it has a certain blocking ability, but the lateral distribution is unstable. Type III: The two sand bodies are vertically superimposed. The interlayer has no thickness, indicating that the upper channel has cut and eroded the lower sand body, presenting a sandstone interface, and the blocking ability is weak.

**Fig. 11:**
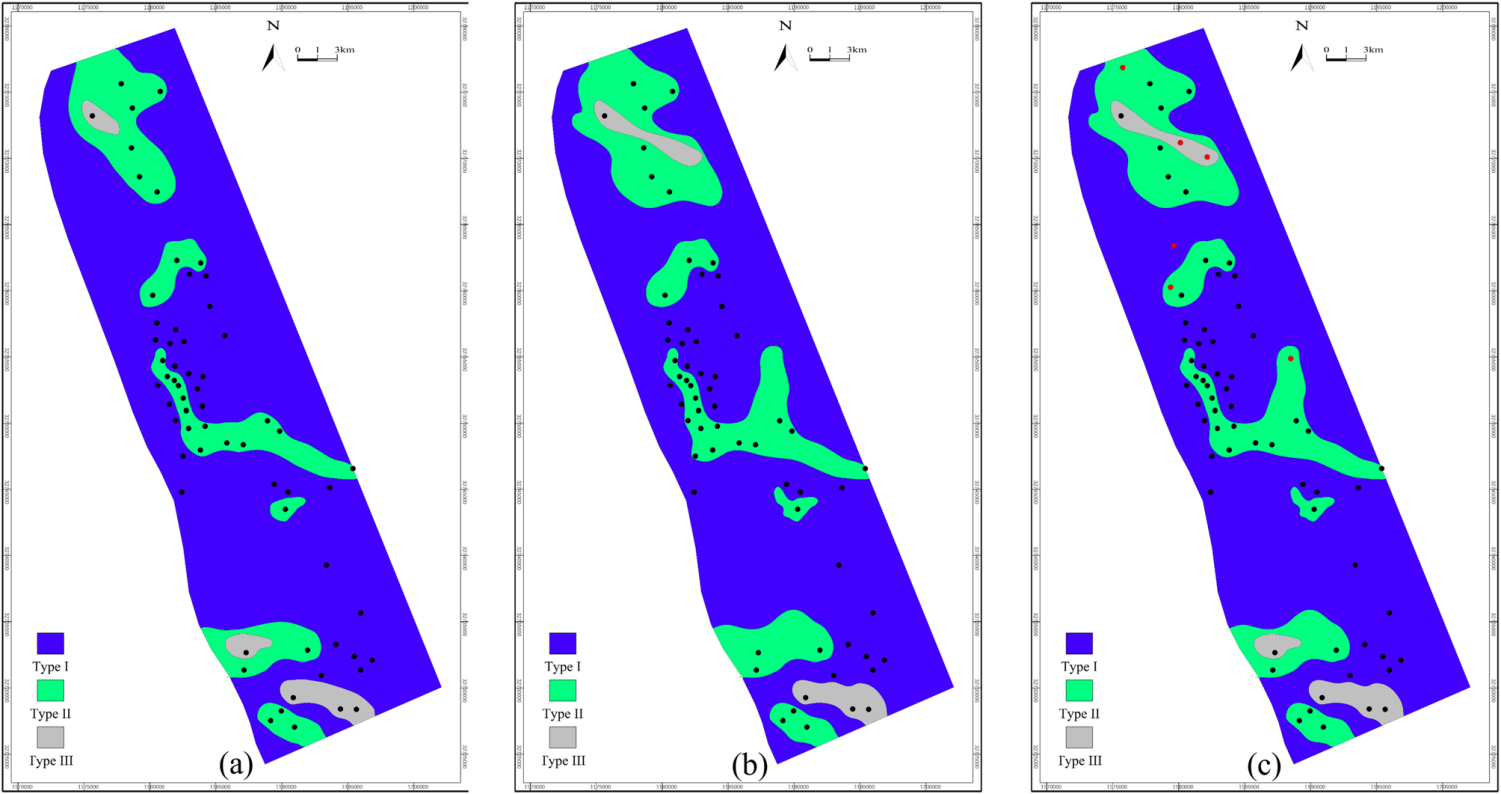
Identification of interlayers. (**a**) Identification of interlayers in wells, (**b**) Interlayers prediction using waveform inversion features, (**c**) Identification of interlayers with the constraint of wells and waveform inversion results. New wells are shown with red points.

Bottom water in the LU_F has a positive effect on maintaining formation energy, but it will lead to premature water breakthrough and rapidly increasing water content when improperly controlled, thus reducing the development performance of bottom water reservoirs. Developed interlayer in formation often makes the vertical connectivity of the reservoir worse and forms an isolated edge-water reservoir. Therefore, it is essential to finely delineate the interlayer using the identification of interlayers in wells (Fig. 11a), the analysis of composite sand-bodies architecture, and waveform inversion features (Fig. 11b). By combining the above features, some bottom-water reservoirs are reinterpreted as a combination of the upper edge-water reservoir and the lower bottom-water reservoir, which are verified by the new drilling wells and shown in Fig. 11c. Take the middle part of wells in the area as an instance, the wells are interpreted as partially developed type II interlayers using only the wells. Continuous type II interlayers, however, are well developed in the middle part which is interpreted using seismic data. The production performance verifies the connectivity of the interlayers without well constraints and should follow the seismic information.

Statistics on the thickness and length of the mud interlayer in the sand bar of this area show that 61% interlayers are around 1 ft thick, 20% are between 1 and 2 ft and 19% are over 2 ft. The lengths of the interlayer are between 2 and 8 km for 30%, and 70% of the interlayer lengths are greater than 8 km. Combined with the interpretation result of seismic waveforms, the updated interpretation result shows that the thickness of interlayer becomes thinner, the length of interlayer increases significantly and lateral variations are dramatic in the area when the tidal strength increases.

### Reservoir modeling

Three-dimensional geological modeling is performed based on fine reservoir description. Integrating geological, seismic, logging, testing, and production data, fine description and analysis of the reservoir is performed to show sedimentary features of oilfields. In detail, taking a single layer or sandstone as the basic unit, the reliability and precision of modeling using comprehensive deterministic modeling with stochastic modeling under the constraint of sedimentary facies can be improved and correctly reveal the geological features of the reservoir.

**Fig. 12:**
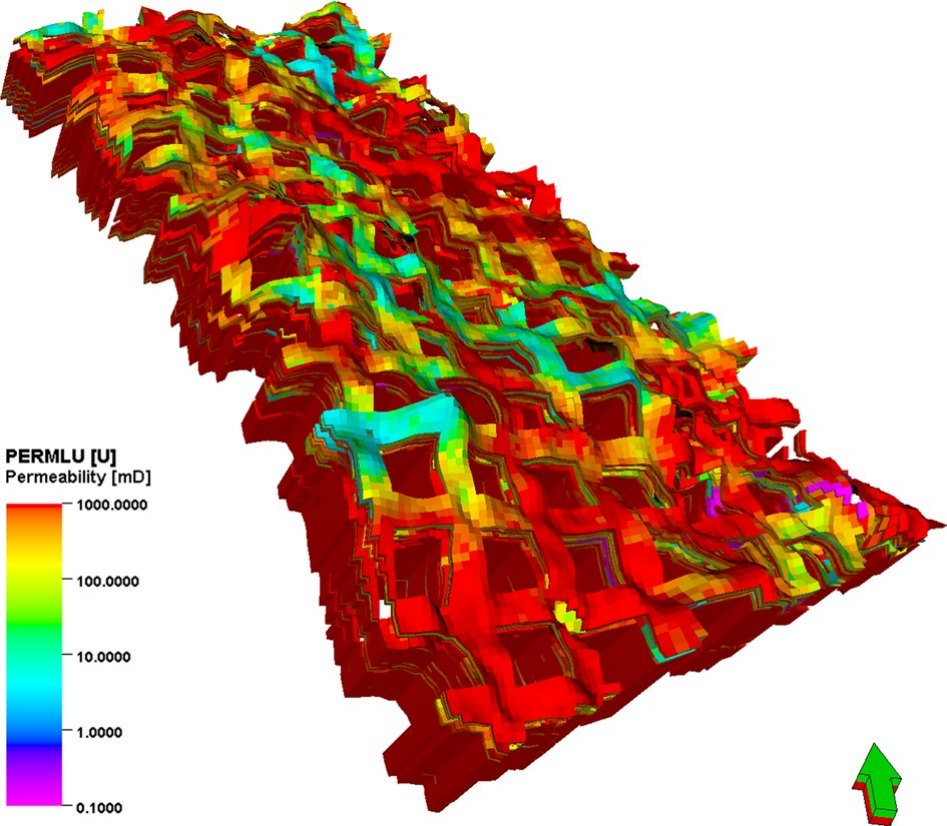
Three-dimensional geological model of permeability.

**Fig. 13:**
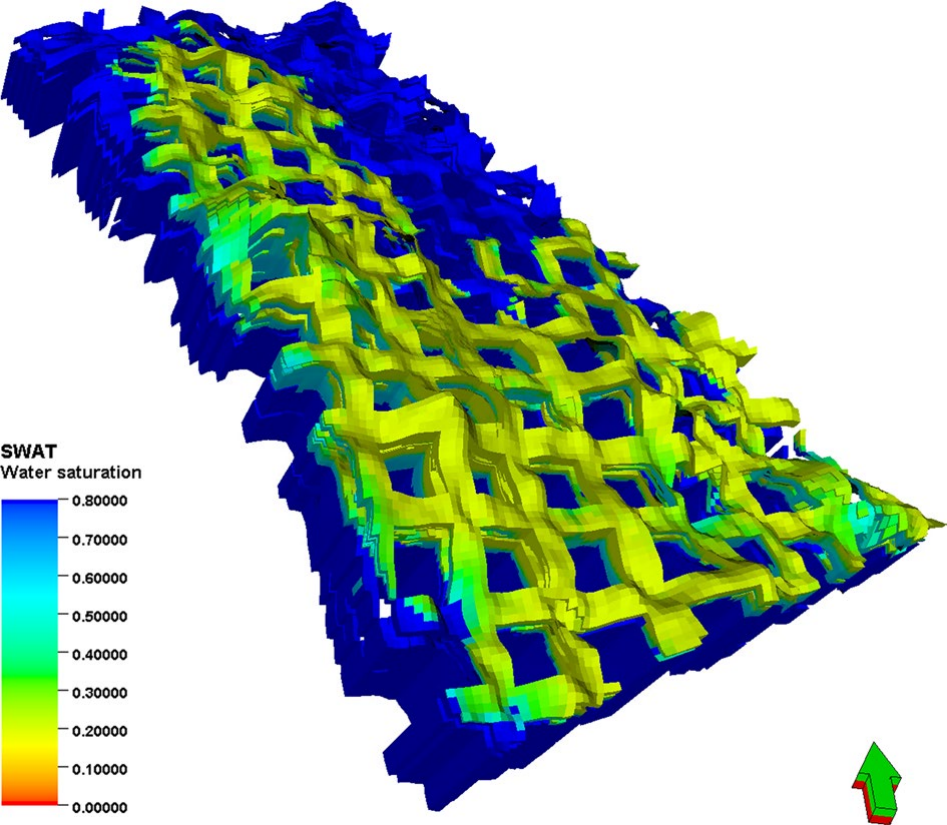
Three-dimensional geological model of water saturation.

Guidelines are presented for each of the following main tasks and modeling scope: (1) Data collection and preparation. To build this kind of model, numerous steps start with gathering the data: Different well log data are collected for building the model, such as GR, density, and resistivity logs. The data exported from Geolog Software includes water saturation, porosity, and permeability. Interpreted horizons with lines that include points of equal value and separate points of higher and lower value, which display depth of a formation. (2) Well correlation. Compares well log data (e.g., GR and resistivity) across multiple wells to map subsurface formations. The resulting well correlation section reveals vertical (depth-related) and lateral (spatial) changes in formation thickness, as well as variations in petrophysical properties such as porosity, permeability, and water saturation. (3) Structural Modeling. The initial step in building the 3D model involves creating a 3D grid, which is a network of horizontal and vertical lines used to represent the three-dimensional geological model. Pillar gridding is a fundamental process for generating the grid that forms the basis of all modeling. The grid is composed of top, mid, and base skeleton grids. For the study area, a three-dimensional grid system of 25 m per grid cell along the X-axis and 25 m per grid cell along the Y-axis is used. The structural modeling involves placing the stratigraphic horizons into the simple grid while considering the grid increment. Zones are built based on well tops from all wells and the available structural map for target formations from 3D seismic data. Each created zone is subdivided into many layers of the same thickness according to the geological deposition of a specific zone. It should be noted that the model of each layer can be accurately established by taking the existing geological understanding data as the conditioned data, due to the limited distribution density of wells in the study area. The length, width, and height of the collected sand bodies can be used to determine the variogram of the tidal sand in the study area. At the same time, the scaling relationships are generally consistent with modern sedimentary features. Under the guidance of modern sedimentary features, variogram analysis is carried out to determine the variogram data of major range, minor range, and vertical range, which provides the basis for facies modeling. Based on the sedimentary facies model, the three-dimensional geological model of porosity is realized by the sequential Gaussian simulation method. Based on the reservoir physical property parameters relationship between porosity and permeability interpreted from the logging, the three-dimensional geological model of permeability is realized by the co-simulation method of porosity as the constraint and shown in Fig. 12. At the same time, the three-dimensional geological model of water saturation is established under the constraint of permeability and shown in Fig. 13. The inversion results, combining the seismic response characteristics with strong trend mappings, can reflect the sandstone continuity without controlled wells, thus making up for the shortcomings of the uncontrolled area without wells in the sedimentary facies and concluding semi-deterministic method which considers seismic data provides greater reliability than stochastic method without the constraint of seismic information.

The porosity of LU is mainly distributed at 16–26%, and the permeability is mainly distributed at 200 × 10^–3^–000 × 10^–3^ μm^2^. According to cut-offs by logging discipline, combining the detail of the model, the function is determined for Net-to-Gross calculation: NTG = if (Phie > 0.1,1, 0), that is mean when porosity is larger than 0.1, the cells are defined as valid cells for reserve calculation. Oil pool distribution is mainly controlled by structure and lithology, and the Oil–Water Contact (OWC) becomes higher from west to east with the structure rising. Due to the strong edge-bottom water energy, the reservoir was severely water flooded at the bottom, and the remaining oil was accumulating at the top in the north area. By comparing the logging information of the new drilling well with the simulated logging information of the reservoir model, the reliability of porosity and permeability models are mainly verified with accuracy reaching 91%.

The outcomes of the study reveal that, within the constraint defined by seismic data-delineated sand boundaries, the predicted well logs in the model exhibit a high degree of consistency with core analysis, achieving a relative error of 7.6% between the verification wells and their respective well log. This represents a significant improvement over the initially interpreted outcomes, suggesting that, in tidal-dominated estuarine reservoirs, the adoption of a seismic data-constrained reservoir modeling approach can effectively enhance the accuracy of the model in areas sparsely populated with wells. Consequently, it offers invaluable guidance for the exploration and development strategies of hydrocarbon-bearing reservoirs situated within tidal-controlled estuaries.

## Discussion

A study on the fine characterization of the tidal-dominated estuary reservoirs was carried out by incorporating seismic data, which described the distribution and properties of subsurface reservoirs. A stable workflow has been implemented to complement the lateral characterization of tidal-dominated estuary sandstones in the study area. Integrating seismic data enhances the certainty of the characterization, although it also introduces inconsistencies due to the fundamentally different properties measured by electrofacies and seismic data, which require expert geological interpretation for effective integration.

The development of interlayers within the sand bar—formed during the slack water period of the tidal cycle, when sediments were deposited following strong tidal disturbances—is indicative of a distinct sedimentary event. From the point of view of sedimentary dynamics, the degree of sediment deposition to the sea is weak when the tidal strength is small, and the sediment deposition to the sea at this time mainly relies on the impact of fluvial sources. This result aligns with previous findings by Tang et al.^36^, who used experiments to simulate tidal channel emergence in the offshore section of the sand bar, and indicates the development of the sand bar has been influenced by the combined action of fluvial and tidal forces during the formation of the tide-dominated estuary. Therefore, the study area conforms to the sedimentation law of modern estuaries.

For sedimentary characterization of tide-dominated estuarine systems, Dalrymple et al.^37^ established the classic sedimentary pattern of the tidal controlled estuary through the investigation of the modern Cobequid estuarine bay. However, new drilled wells have demonstrated inherent limitations in reservoir prediction accuracies when relying solely on well-based electrofacies interpretation^38^, particularly regarding spatial heterogeneity characterization. The seismic facies analysis conducted in this study enhances the reliability of later-stage predictions that cannot be resolved by well data alone. The integration of seismic facies with electrofacies establishes a robust multi-scale characterization framework: seismic attributes provide meso-scale architectural constraints from rock elastic properties, electrofacies offer centimeter-scale vertical resolution form rock radioactive properties. Although no one—to—one correspondence exists between seismic facies and electrofacies since seismic data reflects a combination of geophysical factors, only some of which are directly related to lithofacies, each seismic facies is associated with a unique distribution of electrofacies.

Meanwhile, the study on the sand architecture of the LU layer can provide a strong implication for the distribution of the sandstone and architecture of the tidal-dominated estuary. In tidal-controlled estuary environment, we can find that the geometry of tidal sand bars varies widely, where length-to-width ratios are roughly distributed between 3–15 times based on seismic facies. These values can be incorporated into variogram modeling to enhance spatial predictions and reflect their geological significance. Although their geometric features have strong statistical laws, a wide range of distributions can be noted. Modeling on this basis will inevitably bring a certain degree of uncertainty. The solution to this problem is to further subdivide the geometric features of sand bodies by incorporating more information. In this way, the sedimentary understanding can then be used to guide geological modeling, the uncertainty of the modeling results can be reduced and the accuracy of the simulation results can be improved.

Nowadays, the water cut of the study area reaches 93%, some of the main producing oilfields even reach 95%, and the remaining oil distribution is sparse and scattered. The area without the constraint of wells shows high risk and uncertainty for new proposal wells. Therefore, it is of great significance to integrate newly available data to investigate the remaining oil, which will help us update reservoir understanding and build a reliable reservoir model to predict the distribution of residual oil. Comprehensive reservoir characterization should be quantitatively automated for potential analysis of new well proposal in time. It should be noted that interpretation using a resistivity tool have to be compensated for the effects from the invasion of mud filtrate and borehole conditions, since the interpreted results are only reliable when the influence of borehole and surrounding formations are minimized.

An integrated workflow that combines electrofacies, seismic facies, and sedimentological analysis provides an effective approach to extending highly localized information^34,39,40^. However, these studies focus on fluvial and deltaic deposits, which contrasts with estuarine systems characterized by dual hydrodynamic forces of tidal and wave-driven mixing. This divergence expands the understanding of depositional controls beyond common fluvial and deltaic systems. Also, machine Learning method used in this paper is robust which offers computational efficiency and requires minimal training data, making it advantageous for data-scarce regions.

## Conclusion

In this paper, a study on the fine characterization of tidal-dominated estuary reservoirs is carried out and a geological model is established based on electrofacies and seismic facies under well constraints. By comprehensively analyzing core data, seismic, and well logs, we can find that the LU layer of Napo Formation in Oriente Basin develops tidal estuary sedimentation, which can be further divided into four electrofacies: tidal channel, tidal sand bar, sand flat, and mixed flat. Based on the combination of well logs and seismic data, a reliable fine delineation of a tidal-dominated estuary reservoir is proposed to characterize the distribution of sedimentary facies in the study area. This delineation is constrained by the electrofacies at the location of wells, trend maps of seismic attributes, and facies boundaries of seismic inversion profiles. After that, the analysis of interlayer development in different wells (e.g., LU_T with vertically separated interlayers versus LU_F with stacked sandstone) reveals the significant vertical and lateral heterogeneity of reservoirs. This heterogeneity must be taken into account when modeling reservoirs and predicting oil distributions, as it can significantly impact fluid flow and production strategies. Finally, the use of sedimentary understanding to guide geological modeling reduces modeling uncertainty and improves simulation accuracy. By integrating core data, well logging, and seismic facies analysis, researchers can establish a reliable fine delineation of tidal-dominated estuary reservoirs. This approach enables the quantitative and reliable characterization of subsurface structures and sedimentary characteristics^41^.

## Data Availability

The datasets used and analysed during the current study available from the corresponding author on reasonable request.
